# Behcet's Disease with Intracardiac Thrombus Presenting with Fever of Unknown Etiology

**DOI:** 10.1155/2015/149359

**Published:** 2015-09-03

**Authors:** Sajal Ajmani, Durga Prasanna Misra, Deep Chandh Raja, Namita Mohindra, Vikas Agarwal

**Affiliations:** ^1^Department of Clinical Immunology, Sanjay Gandhi Postgraduate Institute of Medical Sciences, Lucknow 226014, India; ^2^Department of Cardiology, Sanjay Gandhi Postgraduate Institute of Medical Sciences, Lucknow 226014, India; ^3^Department of Radiodiagnosis, Sanjay Gandhi Postgraduate Institute of Medical Sciences, Lucknow 226014, India

## Abstract

A young male was referred to us for evaluation of fever of unknown origin (FUO). He had history of recurrent painful oral ulcers for one year and moderate to high grade fever, pustulopapular rash, and recurrent genital ulcers for 6 months and hemoptysis for 3 days. He was detected to have intracardiac thrombi and pulmonary arterial thrombosis along with underlying Behcet's disease (BD). Patient responded to high dose prednisolone (1 mg/Kg/day) along with monthly parenteral cyclophosphamide therapy. This case highlights the fact that BD is an important cause for pulmonary artery vasculitis with intracardiac thrombus formation, and such patients can present with FUO.

## 1. Introduction

Behcet's disease (BD) is a multisystem inflammatory disease. There is no definitive laboratory test to confirm BD; hence diagnosis is based on clinical features. A number of diagnostic criteria have been proposed for BD. The 1990 International Study Group (ISG) criteria [1] mandate the presence of oral ulcers along with two of the following: recurrent genital ulceration, eye lesion (anterior or posterior uveitis), skin lesions (erythema nodosum, pseudofolliculitis, papulopustular lesions, and acneiform nodules), and positive pathergy test. The International Criteria for Behcet's Disease (ICBD) [2] have a higher sensitivity and provide a weighted score to the various manifestations of BD. Ocular lesions, oral aphthosis, and genital aphthosis are each assigned 2 points, while skin lesions, central nervous system involvement, and vascular manifestations score 1 point each. The pathergy test, when used, scores 1 point. A score of at least 4 points is classified as BD.

Despite the multisystem involvement, cardiac pathology is unusual. Pancarditis, acute myocardial infarction, conduction system disturbances, and valvular disease have all been described [3]. Intracardiac thrombus formation is very uncommon. We present a case of a young male who was referred to us as fever of unknown origin (FUO) and was subsequently diagnosed to have BD with pulmonary artery vasculitis and intracardiac thrombus.

## 2. Case Presentation

**Figure 1:**
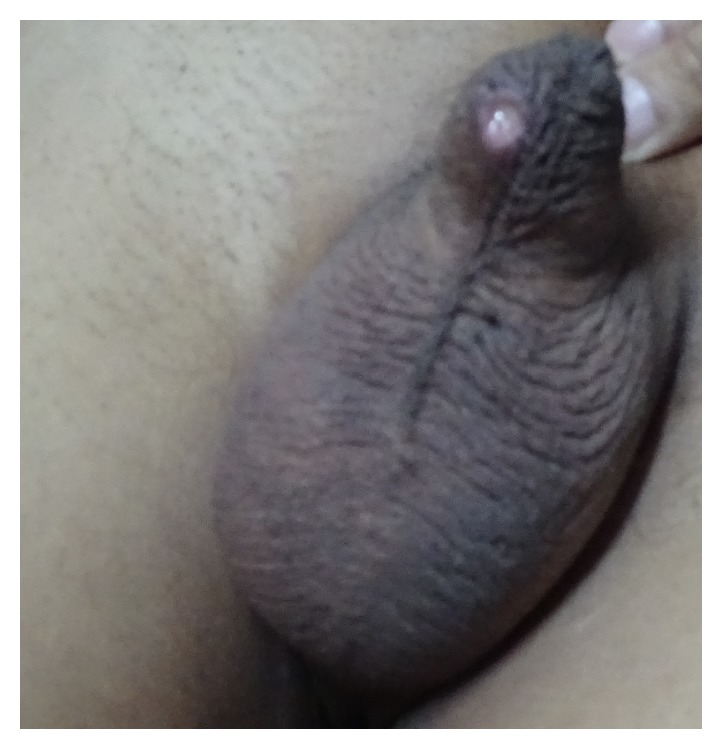
Ulcer in the ventral surface of the shaft of the penis.

A 36-year-old male was referred to us for evaluation of FUO. He had history of recurrent painful oral ulcers, 4 episodes in the past 1 year, each time lasting for around 2 months. For the past 6 months, he complained of daily high grade fever, with chills and rigors, documented to be around 39.4–40°C. In addition to fever, recurrent crops of erythematous papulopustular rash over the abdomen, forearm, and thigh and recurrent genital ulcers on the scrotum and shaft of penis were reported for last 6 months. For the past month, he had right lower chest pain, worse on coughing, associated with shortness of breath which had progressed over the month to the extent that he had shortness of breath even on day-to-day activities. He had history of streaky hemoptysis for the past 3 days. He also had history of significant weight loss, night sweats, and loss of appetite. There was no history of joint pain, abdominal pain, diarrhea or blood mixed stools, lymph node enlargement, recent onset hypertension, oliguria, hematuria, edema over feet or periorbital edema, altered sensorium, headache, stroke, and thrombosis in the past. There was no family history of stroke in young or deep venous thrombosis to suggest a hypercoagulable state. He was in a stable monogamous relationship for a number of years. He had not received blood transfusions in the past or ever abused intravenous drugs. There was no history of contact with tuberculosis. He had received multiple courses of broad spectrum antibiotics elsewhere without any relief. On examination pulse rate was 90/min, blood pressure was 126/70 mm Hg in the right upper limb, and respiratory rate was 22/min. General physical examination revealed severe pallor, oral ulcers over tongue and cheek, small, 0.5 × 0.5 cm with clean base. A genital ulcer (Figure 1) measuring 2 × 2 cm was present over the penile shaft ventral surface with erythematous border and showing signs of healing with healthy granulation tissue at the base. There were hyperpigmented macules over back and abdomen, suggestive of healed rash. Rest of the general physical and systemic examinations were unremarkable.

**Figure 2:**
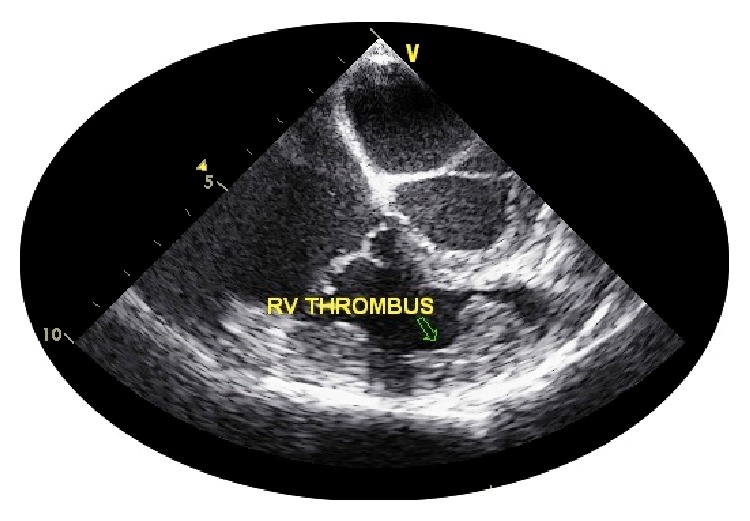
Transoesophageal Echocardiographic (TOE) image at midoesophagus (short axis) showing the right ventricular (RV) thrombus (arrow mark) along the free wall of RV, extending from just below tricuspid valve to the right ventricular outflow beneath the pulmonary valve.

Hematological investigations revealed a normocytic anemia (hemoglobin 8.7 g/dL), total leucocyte count was 10,200/mm^3^ (neutrophils 72%, lymphocytes 28%), platelet count was 327000/mm^3^, normal renal (blood urea 16 mg/dL, serum creatinine 1 mg/dL) and liver function tests (bilirubin 0.5 mg/dL, aspartate aminotransferase 37 U/L, alanine aminotransferase 28 U/L, alkaline phosphatase 155 U/L, serum protein 6.7 g/dL, and serum albumin 3.6 g/dL). He had markedly elevated acute phase reactants (ESR by Westergren's method 70 mm/hr, C-reactive protein 9.25 mg/dL, normal <0.6 mg/dL). Computed tomography (CT) of chest and abdomen revealed minimal effusion in right pleural cavity and mild hepatosplenomegaly and thickening of the distal ileum and caecum. Serology for HBsAg, anti-HCV, and HIV-ELISA were negative. Three blood cultures did not grow any microorganisms. Two-dimensional transthoracic and transesophageal echocardiography (Figure 2) revealed a thrombus in right ventricle with broad base and extending from the tricuspid valve to the pulmonary valve. Mantoux (10 TU) and pathergy tests were negative. CT pulmonary angiography was done in view of hemoptysis and revealed multiple peripheral pulmonary artery thrombi.

Our patient fulfilled both ISG and ICBD criteria (orogenital ulcers, skin lesions, and vascular manifestations—hence scoring 6 points, fulfilling ICBD criteria for the classification of BD) and hence was diagnosed as having BD. Mild pleural effusion [4] and bowel thickening akin to Crohn's disease [5] have been well described in BD and hence were attributed to the primary disease. In view of the fact that he had been extensively evaluated prior to presenting to us and no cause of fever was identified in spite of multiple prior hospital visits and admissions, he was labeled as FUO, subsequently identified to be due to cardiac and pulmonary artery thrombi [6]. He was started on colchicine, 1 mg/kg of prednisolone, and aspirin and given 1st dose of cyclophosphamide (he has been planned for 6 doses of monthly cyclophosphamide, 750 mg/m^2^). With therapy, all his symptoms improved in a week and he was discharged in stable condition. He was asymptomatic when contacted over the phone a month later and was due for the second dose of cyclophosphamide.

## 3. Discussion

Although it was possible to suspect BD from the first instance itself due to presence of oral and genital ulcers, fever is considered to be an uncommon manifestation of BD. A study in 500 patients of BD reported that 22% of patients reported a history of febrile episodes. The presence of fever was found to be strongly associated with vascular, neurological, or joint involvement [5]. In our patient, subsequent investigation revealed presence of intracardiac and pulmonary artery thrombi which were the cause of fever.

Intracardiac thrombosis in BD is rare. A review of literature done in 2000 [7] reported only 24 cases in literature till then. Intracardiac thrombus can occur not only in adults, but also in children with BD [8]. It is a serious complication with poor prognosis and often occurs in association with pulmonary artery aneurysm (42%), pulmonary thromboembolism (52%), and venous thrombosis (56%). At the time of detection of intracardiac thrombus, fever, hemoptysis, dyspnea, and cough are the predominant symptoms (seen in 52%, 48%, 44%, and 20% of patients, resp.). Young men (usually third decade) are most often affected, and the right heart is the most frequent site of involvement. Because intracardiac thrombus is tightly attached to the endocardium or myocardium, thromboembolism from the cardiac cavity seems to be relatively uncommon. The pulmonary abnormalities have been seen to resolve in some cases after administration of immunosuppressive treatment rather than anticoagulation [9–11]. It is, therefore, likely that the pulmonary vascular involvement is a result of in situ inflammation rather than embolization. Patients of BD with intracardiac thrombus usually have prominent constitutional symptoms such as fever, due to which it is difficult to differentiate it from infective endocarditis and atrial myxoma. The pathologic mechanism of thrombus formation in BD is believed to be endothelial cell ischemia or disruption leading to platelet aggregation [12]. Decreased release of antithrombotic tissue plasminogen activator from blood vessels has also been reported [13].

Treatment of intracardiac thrombus is unclear. Some patients have been treated surgically with removal of thrombus. In the small number of cases published in literature, patients treated with medical therapy tend to do better than those treated surgically [14]. Most patients have been treated with colchicine and steroids. Some have been treated with cyclophosphamide in addition and have shown good response. Anticoagulation in patients with cardiac thrombus is controversial since these have a low chance of thromboembolism and a high risk of bleeding in presence of pulmonary artery aneurysm, which can be life threatening. Often, resolution of the thrombus has been seen with just immunosuppression with or without antiplatelet agent [7]. Our patient responded to treatment with steroids, cyclophosphamide, and aspirin without anticoagulation.

In conclusion, BD should be kept in differential diagnosis in a young male with intracardiac thrombus. Such patients usually have marked constitutional symptoms and can present with FUO. It is also important to look for vascular, neurological, and joint involvement in patients with BD if they have fever. A keen clinical acumen is essential to diagnose these patients in a timely manner and institute life-saving immunosuppression at the earliest.
